# P38 Mitogen-Activated Protein Kinase Inhibitor, FR167653, Inhibits Parathyroid Hormone Related Protein-Induced Osteoclastogenesis and Bone Resorption

**DOI:** 10.1371/journal.pone.0023199

**Published:** 2011-08-23

**Authors:** Huiren Tao, Mina Okamoto, Masataka Nishikawa, Hideki Yoshikawa, Akira Myoui

**Affiliations:** 1 Department of Orthopedics, Osaka University Graduate School of Medicine, Osaka, Japan; 2 Department of Orthopedics, Xijing Hospital, Fourth Military Medical University, Xi'an, China; National Institutes of Health, United States of America

## Abstract

p38 mitogen-activated protein kinase (MAPK) acts downstream in the signaling pathway that includes receptor activator of NF-κB (RANK), a powerful inducer of osteoclast formation and activation. We investigated the role of p38 MAPK in parathyroid hormone related protein (PTHrP)-induced osteoclastogenesis *in vitro* and PTHrP-induced bone resorption *in vivo*. The ability of FR167653 to inhibit osteoclast formation was evaluated by counting the number of tartrate-resistant acid phosphatase positive multinucleated cells (TRAP-positive MNCs) in *in vitro* osteoclastgenesis assays. Its mechanisms were evaluated by detecting the expression level of c-Fos and nuclear factor of activated T cells c1 (NFATc1) in bone marrow macrophages(BMMs) stimulated with sRANKL and M-CSF, and by detecting the expression level of osteoprotegerin (OPG) and RANKL in bone marrow stromal cells stimulated with PTHrP in the presence of FR167653. The function of FR167653 on bone resorption was assessed by measuring the bone resorption area radiographically and by counting osteoclast number per unit bone tissue area in calvaria in a mouse model of bone resorption by injecting PTHrP subcutaneously onto calvaria. Whole blood ionized calcium levels were also recorded. FR167653 inhibited PTHrP-induced osteoclast formation and PTHrP-induced c-Fos and NFATc1 expression in bone marrow macrophages, but not the expression levels of RANKL and OPG in primary bone marrow stromal cells treated by PTHrP. Furthermore, bone resorption area and osteoclast number in vivo were significantly decreased by the treatment of FR167653. Systemic hypercalcemia was also partially inhibited. Inhibition of p38 MAPK by FR167653 blocks PTHrP-induced osteoclastogenesis *in vitro* and PTHrP-induced bone resorption *in vivo*, suggesting that the p38 MAPK signaling pathway plays a fundamental role in PTHrP-induced osteoclastic bone resorption.

## Introduction

Parathyroid hormone related protein (PTHrP), a potent stimulator of osteoclastic bone resorption, was first identified as a causative factor for humoral hypercalcemia of malignancy [1]. A number of clinicopathological and experimental studies have shown that cancer cell-derived PTHrP promotes osteoclastic bone resorption and contributes to the development and progression of cancer metastasis to bone [2]–[5]. PTHrP stimulates osteoclastogenesis by acting on osteoblasts and/or bone marrow stromal cells to increase receptor activator of NF-κB ligand (RANKL) expression and reduce osteoprotegerin (OPG) expression, not by acting directly on osteoclast precursors [6]. Osteoclast precursors that express RANK, a tumor necrosis factor (TNF) receptor family member, recognize RANKL and differentiate into osteoclasts in the presence of macrophage/monocyte colony stimulating factor (M-CSF). OPG is a soluble decoy receptor for RANKL and has the ability to inhibit osteoclastogenesis. Therefore, a relative increase of RANKL versus OPG expression by PTHrP activates osteoclastic bone resorption [7]–[12].

Additionally, a body of evidence suggested that p38 mitogen-activated protein kinase (MAPK) is downstream of the RANK signaling pathway and plays an important role in osteoclast differentiation. The expression of dominant-negative forms of p38 MAPK in RAW264.7 cells inhibited RANKL-induced differentiation of these cells into osteoclasts [13]. Li et al used pyridinylimidazole SB203580, a specific inhibitor of p38 MAPK, to study p38 MAPK function and demonstrated that p38 MAPK is required for osteoclast differentiation [14].

FR167653 was first discovered as a potent inhibitor of TNFα and interleukin (IL)-1β production in lipopolysaccharide-stimulated human monocytes and phytohemagglutinin-M-stimulated human lymphocytes [15], [16]. FR167653 inhibits the activation of p38 MAPK by suppressing the phosphorylation of p38 MAPK, preferentially affecting its α isoform, but not the γ isoform [17]–[19]. FR167653 is effective in treating inflammation, relieving trauma and ischemia-reperfusion injury *in vivo* [20]–[22]. We previously reported that M-CSF-dependent sRANKL- and TNFα-induced osteoclast formation in primary bone marrow cells, and collagen-induced arthritis in rats were inhibited by FR167653 [23]. However, the relationship of p38 MAPK and PTHrP in osteoclastogenesis and bone resorption is still unclear.

Here we investigate the role of FR167653 on PTHrP-induced osteoclastogenesis, local bone resorption, and hypercalcemia. We found that FR167653 not only blocked osteoclast differentiation induced both by PTHrP and sRANKL with down regulation of c-Fos and NFATc1 in bone marrow macrophages without affecting RANKL and OPG expression in bone marrow stromal cells, but also alleviated bone resorption induced by PTHrP with partial reduction of hypercalcemia in vivo.

## Materials and Methods

### Animal and reagents

All animal experiments in this study strictly followed a protocol approved by the Institutional Animal Care and Use Committee of Osaka University (approval number: 338). Six-week-old male ddy mice and four- to eight-week-old male BDF1 mice were purchased from Japan SLC (Hamamatsu, Japan) and Oriental Yeast (Tokyo, Japan), respectively. Human recombinant PTHrP (1–34) was purchased from Peptide Institute (Osaka, Japan). M-CSF and soluble recombinant RANKL were purchased from PeproTeck EC (London, UK). FR167653 was provided by Asteras Pharma (Osaka, Japan). c-Fos and actin antibodies were purchased from Cell Signaling Technology (Danvers, USA). NFATc1 antibody was bought from BD Biosciences Pharmingen (Franklin Lakes, USA).

### Primary bone marrow cell culture

Bone marrow cells were collected by removing the tibias and femurs from ddy mice and flushing the bone cavity with serum-free alpha-minimum essential medium (αMEM; Invitrogen, Carlsbad, CA). A sample of 7.5×10^5^ cells in a volume of 0.5 ml/well were cultured in αMEM supplemented with 10% fetal bovine serum (FBS; Equitech-Bio, Kerrville, TX) in the presence of PTHrP (45 ng/ml). Some cultures received an additional treatment of 10 µM, 1 µM or 0.1 µM FR167653. The medium was changed every two days, replacing half of the medium with 0.25 ml fresh medium containing 90 ng/ml PTHrP and 20 µM, 2 µM and 0.2 µM FR167653. After six days of culture, adherent cells were stained for tartrate-resistant acid phosphatase (TRAP), an osteoclast marker enzyme, using a TRAP staining kit (Hokudo, Sapporo, Japan). TRAP-positive multinucleated cells (MNCs) with three or more nuclei were counted as osteoclasts.

### M-CSF-dependent bone marrow macrophage (MDBMMs) culture

M-CSF-dependent bone marrow macrophages (MDBMMs) were cultured as described previously [23]–[25]. Briefly, bone marrow cells (5×10^6^) were cultured in the αMEM supplemented with 10% FBS, and M-CSF at the concentration of 100 ng/ml in 100 mm dishes for three days. Then the cells were washed and harvested with 0.02% ethylene diamine tetra-acetate (EDTA) in phosphate-buffered saline, and seeded at a density of 3×10^5^ into 100-mm culture dishes in the presence of M-CSF (100 ng/ml). After three more days of culture, the adherent cells were considered as MDBMMs.

For osteoclast culture, MDBMMs were digested with 0.25% Trypsin-EDTA (Invitrogen) and 1×10^4^/well cells were re-plated into 48-well plates in the presence of sRANKL (100 ng/ml) and M-CSF (100 ng/ml). Some cultures were treated with FR167653. After five more days of culture, adherent cells were fixed and stained for TRAP. TRAP-positive MNCs containing three or more nuclei were counted as osteoclasts.

For the detection of the c-Fos and NFATc1expression, MDBMMs were treated by each concentration of FR167653 for 1 hour. Then, sRANKL and M-CSF were added. After another 24 or 48 hour culture, the cells were collected for RNA extraction and protein extraction.

### Real-time polymerase chain reaction

Total RNA was extracted from bone marrow cells (for genes of OPG and RANKL) or bone marrow macrophages (for genes of c-fos and NFATc1) by using the RNeasy Mini Kit (Qiagen, Valencia, CA). RNase-free DNase Set (Qiagen) was used to remove the residual DNA. Five micrograms of RNA were reverse transcribed into cDNA with Ready-To-Go You-Prime First-Strand Beads (Amersham Bioscience, Piscataway, NJ). In order to reduce pipetting error, the final volume of cDNA was reached by adding four volumes RNase-free distilled water to the original cDNA. The resultant product was subjected to real-time PCR.

Real-time PCR was performed in a Light Cycler (Roche Applied Science, Indianapolis, IN) with Light cycler-DNA Master SYBR Green I (Roche Applied Science) and were performed according to the standard protocol recommended by the manufacturer. Reverse transcription was followed by 40 cycles of PCR (denaturation at 95°C for 10 min addition of 1-second at 94°C, 5-second annealing at 58, 60, 61, 59 and 58°C for OPG, RANKL, c-fos, NFATc1 and GAPDH, respecticvely, and 10- second extension at 72°C, and each of optimal fluorescence measurement temperature for 1 second as the amplification and quantification programs, 60–99°C with a heating rate of 0.1°C per second as the melting curve program, and finally, cooling to 40°C.

The primer sequences are as follows:

RANKL: sense: 5′-CAC CAT CAG CTG AAG ATA GT-3′
  antisense: 5′-CCA AGA TCT CTA ACA TGA CG-3′
OPG: sense: 5′-TGGGACCAAAGTGAATGCCGAGA-3′
  antisense: 5′-AGCTGCTCTGTGGTGAGGTTC-3′
c-fos: sense: 5′-CTGGTGCAGCCCACTCTGGT-3′
  antisense: 5′- CTTTCAGCAGATTGGCAATC-3′
NFATc1: sense: 5′- CAACGCCCTGACCACCGATA-3′
  antisense: 5′- GGCTGCCTTCCGTCTCATAG-3′
GAPDH: sense: 5′-ACCACAGTCCATGCCATCAC-3′
  antisense: 5′- TCCACCACCCTGTTGCTGTA-3′

Semiquantitative RANKL, OPG, c-fos and NFATc1 expression levels were determined by normalizing with GAPDH expression level.

### Western blotting

Cells were washed twice with ice-cold PBS and lysed with a buffer containing 20 mM Tris–HCl, 150 mM NaCl, 1% riton X-100, and inhibitors for proteases and phosphatases [25]. Cell lysates were centrifuged at 10,000 g for 20 min and the supernatants were collected. Twenty micrograms of cellular proteins was resolved by SDS–PAGE and transferred onto nitrocellulose membranes. Membranes were blocked with 5% skim milk in Tris-buffered saline containing 0.1% Tween 20 for 1 h and incubated overnight at 4°C with primary antibodies. Membranes were washed, incubated with appropriate secondary antibodies conjugated to horseradish peroxidase for 1 h, and developed using a chemiluminescence system. Relative protein expression was calculated from band densities obtained in three experiments using UMax PowerLook 1100 scanner (Taiwan) and TotalLab gel image analysis software (Nonlinear Dynamics, New England, UK).

### PTHrP-induced bone resorption in an animal model

**Figure 1:**
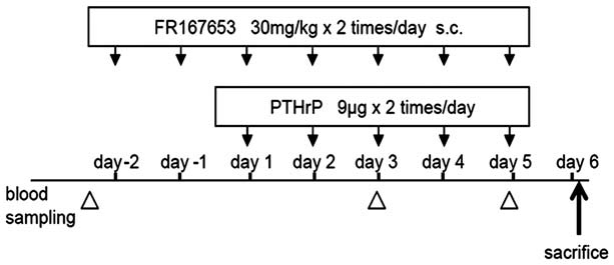
Animal model and treatment protocol. The PTHrP-induced bone resorption animal model was established by injecting PTHrP (18 µg/day) onto the dorsal surface of the mice calvaria daily for five days (day 1 to 5). Animals in the treatment (n = 7) or the control group (n = 5) were injected with FR167653 (30 mg/kg twice daily) or distilled water, respectively, daily for seven days, initiating from two days before PTHrP injection. 50 µl of whole blood was collected retro-orbitally before treatment, on day three, and on day five to determine whole-blood ionized calcium levels. Mice were sacrificed on day six, 12 hours after the last injection of PTHrP.

Male BDF1 mice aged four weeks were injected daily for five days (from day one to five) with PTHrP (18 µg/day; Figure 1). PTHrP was injected into the subcutaneous tissue over the left side of the calvaria twice daily in a volume of 10 µl as described previously [26], [27]. This delivery method produces an exaggerated resorptive response in the calvarial bone, as well as a systemic effect as indicated by hypercalcemia. Animals were housed in an accredited facility and all procedures used in the animal experiments complied with the standards described in the Osaka University Medical School Guidelines for the Care and Use of Laboratory Animal.

### Treatment Protocol

All animals were additionally treated with FR167653 or distilled water, injected subcutaneously on the mice back as in the protocol shown in Figure 1. Treatment was initiated two days before PTHrP injection.

Before treatment, 50 µl of whole blood was collected retro-orbitally into heparinized capillary tubes (Radiometer, Copenhagen, Denmark) and basal whole-blood ionized calcium levels were determined with a calcium level analyzer (model ABL505, Radiometer; n = 3 from treatment group and control group, respectively). The treatment group (n = 7) was treated with 30 mg/kg of FR167653 twice daily while the control group (n = 5) was treated with distilled water for seven days (including pretreatment). At day three and five, blood samples were taken three hours after injection of PTHrP retro-orbitally to determine the levels of hypercalcemia. Mice were killed on day six, which was 12 hours after the last injection of PTHrP.

### Quantitative analysis of bone resorption

Calvaria were retrieved from sacrificed mice and immersed into neutral buffered formalin. Radiographs were taken with the MX-20 Specimen Radiography System (Faxitron X-ray, Wheeling, IL). The images were scanned into a computer with a flatbed scanner and saved in RGB color format. The quantitative analysis of calvarial bone resorption was processed with an image analysis software package, WinROOF (Mitani, Fukui, Japan). The thresholds were determined by clicking five typical bone resorptive areas.

After decalcification, the anterior and posterior portions of the calvaria were removed just anterior to coronal suture and posterior to lambdoid suture, respectively. The left part of the calvaria was cut into three strips, embedded in paraffin and sectioned. Sections were stained to detect TRAP activity and counterstained with hematoxylin.

Histomorphormetic analysis was performed on the left side of the calvaria to determine the number of osteoclasts present in the bone. The area measured consisted of six visual fields extending from sagittal suture toward the lateral muscle attachment and included all of the bone and marrow spaces. PTHrP-treated calvaria showed a distinct dorsal periosteal pattern of resorption and endosteal resorption. Thus, the osteoclast number represented the sum of periosteal and endosteal measurements. Results were recorded as osteoclast number per unit bone tissue area measured (OcN-BTA). All measurements were made by tracing the section image onto a digitizing platen with the aid of a cameral lucida attachment and WinROOF image analysis software.

### Statistical analysis

All analyses were reported as mean ±S.D. Statistical significance was evaluated by Student's *t*-test or one factor analysis of variance (ANOVA) followed by a Tukey-Kramer post test using a statistical software package, JMP (SAS institute, Cary, NC).

## Results

### FR167653 inhibits Osteoclast formation in two different cell culture systems

**Figure 2:**
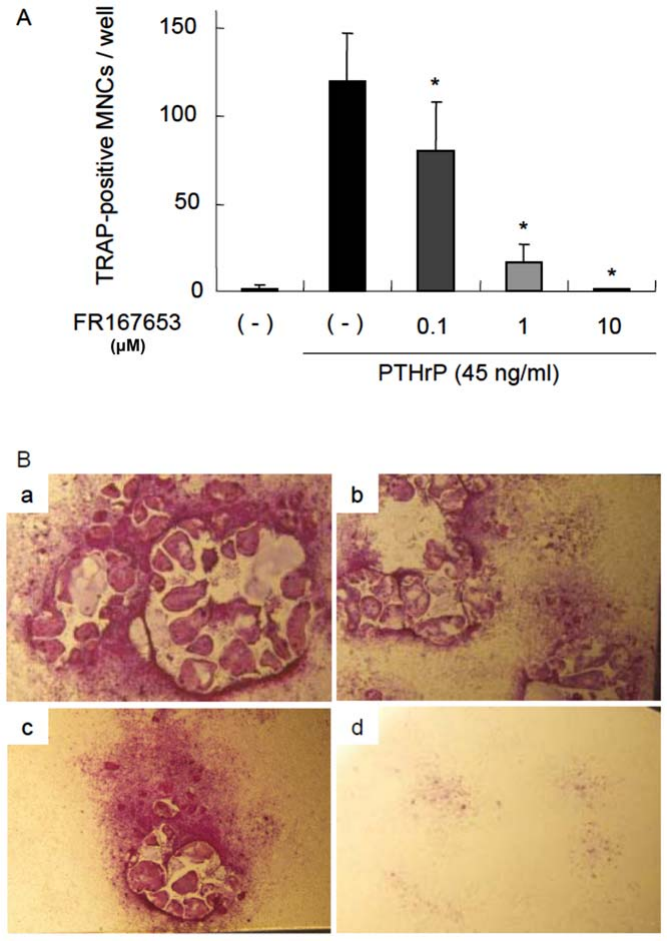
FR167653 inhibits TRAP-positive osteoclast formation in PTHrP-treated bone marrow cells. A. Bone marrow cells were treated with PTHrP (45 ng/ml) and FR167653 for six days. TRAP-positive MNCs with three or more nuclei were counted as osteoclasts. n = 9. *: p<0.001 versus the group treated only with PTHrP. B. TRAP staining for bone marrow cells stimulated with PTHrP (45 ng/ml). a: no treatment. b: FR167653 (0.1 µM). c: FR167653 (1 µM). d: FR167653 (10 µM). TRAP-positive MNCs appear as red cells with clear peripheries.

To determine whether FR167653 blocks osteoclastogenesis *in vitro*, we investigated osteoclastogenesis in a primary bone marrow cells stimulated by PTHrP. As expected, TRAP-positive MNCs (osteoclasts) was formed in primary bone marrow cultures treated with PTHrP. As shown in Figure 2, osteoclast formation induced by PTHrP at day six was inhibited by addition of FR167653 in a dose-dependent manner. At the concentration of 10 µM, FR167653 nearly eliminated osteoclast formation.

**Figure 3:**
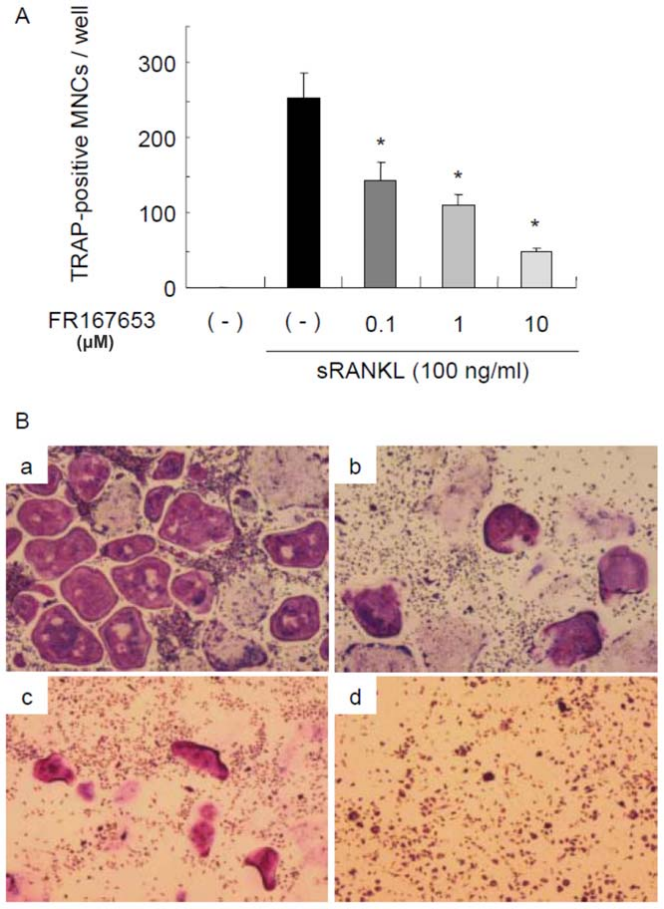
Effects of FR167653 on osteoclast formation in M-CSF-dependent bone marrow macrophages treated with sRANKL. A. Osteoclast formation assay using M-CSF-dependent bone marrow macrophages (MDBMMs) prepared as described in the Materials and Methods section. TRAP-positive MNCs with three or more nuclei were counted as osteoclasts. n = 6. *: p<0.001 versus the group treated only with sRANKL. B. TRAP staining for MDBMMs stimulated by sRANKL (100 ng/ml). a: No treatment. b: FR167653 (0.1 µM). c: FR167653 (1 µM). d: FR167653 (10 µM).

Unlike bone marrow cells, MDBMM cells are a homogeneous bone marrow macrophage population with a contamination by stromal cells of less than 1 in 1,000 cells. As shown in Figure 3, MDBMM cells formed TRAP-positive osteoclasts when treated with RANKL together with M-CSF even in the absence of stromal cells. FR167653 added to the cultures significantly inhibited RANKL-induced osteoclast formation in a dose-dependent manner. These data suggest that FR167653 acts directly on osteoclast precursors to inhibit osteoclast formation.

### OPG and RANKL expression levels in bone marrow cells were not affected by FR167653 treatment

To further elucidate the mechanism of FR167653 inhibition of PTHrP-induced osteoclastogenesis, we examined the effects of FR167653 on the OPG and RANKL expression in bone marrow stromal cells.

**Figure 4:**
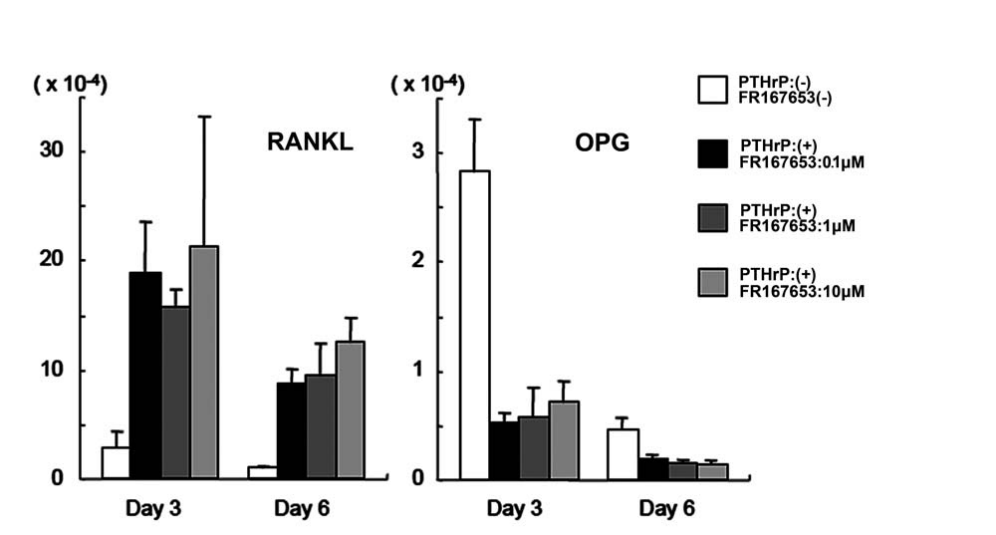
FR167653 does not affect OPG or RANKL expression by PTHrP-treated stromal cells. Bone marrow stromal cells were seeded into six-well plates and treated with PTHrP (90 ng/ml) and FR167653 at various concentrations for three and six days. Real-time PCR was used to determine the OPG or RANKL gene expression levels. The results are shown as mean ±S.D. of three independent experiments. Expression of RANKL and OPG mRNA was increased or decreased, respectively, within three days of PTHrP treatment, and lasted 6 days post-treatment. The expression level of RANKL and OPG did not changed by adding 1 µM or 10 µM FR167653 (*P*>0.05).

Bone marrow stromal cells were obtained by culturing primary bone marrow cells for ten days. Almost all non-adherent or loosely attached hematopoietic cells in the primary bone marrow cell cultures were removed by pipetting each time when the medium was changed. Expression of RANKL and OPG mRNA in bone marrow stromal cells was increased or decreased, respectively, within three days of PTHrP treatment. The increased expression even lasted 6 days post-treatment. Even at a dose of 10 µM, FR167653 has no detectable effect on the PTHrP-mediated expression of RANKL and OPG mRNA (Figure 4). Taken together, these results further suggest that FR167653 acts directly on osteoclast precursors (rather than on stromal cells or osteoblasts) in its role as an inhibitor of osteoclast formation.

### Expression of c-Fos and NFATc1 in osteoclast precursors (MDBMMs) were inhibited by FR167653

**Figure 5:**
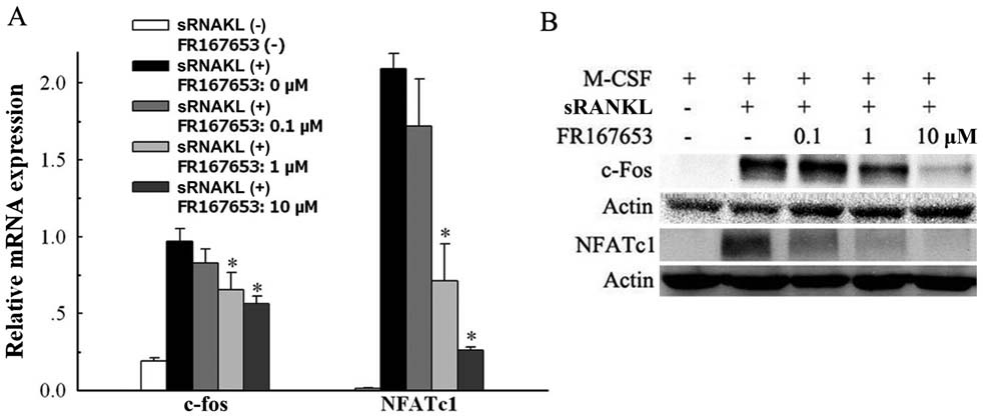
The effects of FR167653 on sRANKL-induced c-Fos and NFATc1 expression level in M-CSF-dependent bone marrow macrophages (MDBMMs). Bone marrow macrophages were treated with 100 ng/ml M-CSF and 100 ng/ml sRANKL without or with FR67653 at the indicated concentration. After 24 h incubation, cells were harvested and subjected to RT-PCR (A). For western blot analysis (B), the cells were incubated for 48 h. Expression of c-Fos and NFATc1 in both mRNA and protein levels were increased following the sRANKL treatment. This enhancement was inhibited by FR167653 in a dose dependent manner. The results are shown as mean ±S.D. of three independent experiments. *: p<0.05 versus the group treated only with sRANKL.

In order to investigate the mechanism of FR167653 on osteoclast formation in osteocalst precursor cells, we detected the expression of c-Fos and NFATc1 in bone marrow macrophage cells by western blotting and RT-PCR following the FR167653 treatments. As shown in Figure 5, the mRNA expression levels of c-fos and NFATc1 were dramatically increased when treated with sRANKL and these effects were inhibited by FR167653 in a dose-dependent manner(p<0.05; Figure 5A). Similarly, c-Fos and NFATc1 protein levels were decreased in the presence of FR167653 dose-dependently (Figure 5B).

### FR167653 reduces PTHrP-induced bone resorption in vivo

**Figure 6:**
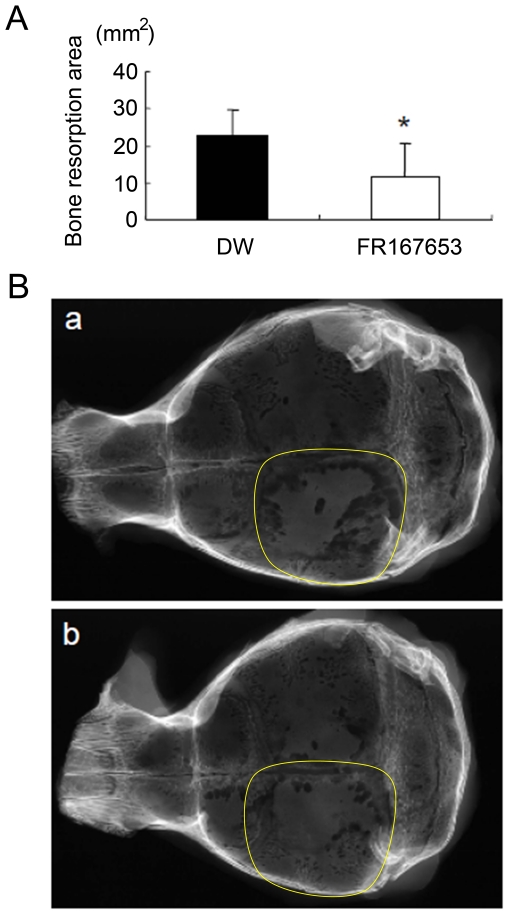
The effect of FR167653 on PTHrP-induced bone resorption. The calvarial bone resorption was measured radiographically on the left side (area marked by yellow line) where treated by direct subcutaneous injection of PTHrP. A. Treatment by FR167653 reduced the bone resorption area (*: P<0.005). DW:distilled water. B. Representative X-rays of mouse calvaria with PTHrP-induced bone resorption. a: control group (treated with DW). b: FR167653 treatment group.

**Figure 7:**
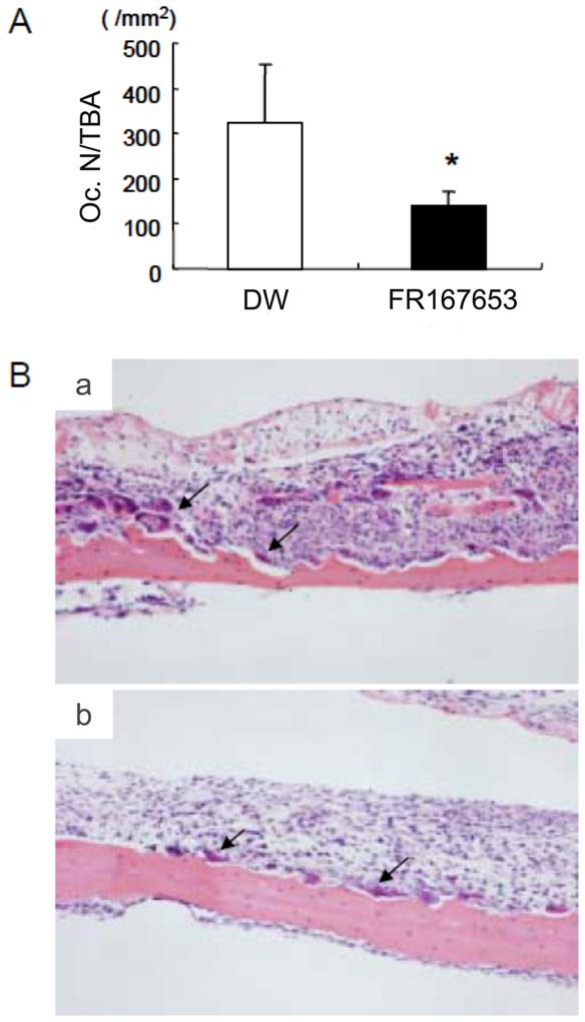
The effects of FR167653 on osteoclast number in calvaria from PTHrP-treated animals. A. Osteoclast number per unit bone area (OcN-BTA) was measured histologically as described in the Materials and Methods section. Treatment of FR167653 reduces the number of osteoclasts (*: P<0.005). B. TRAP staining and counterstain with hematoxylin on calvaria sections. a: Histological section of animals treated with DW. b: Histological section of animals treated with FR167653. Osteoclasts are indicated by arrowheads.

We next examined the effects of FR167653 on PTHrP-induced bone resorption *in vivo*. Animal models of bone resorption were established by direct injection of PTHrP onto the dorsal surface of mice calvaria. Radiographic evidence of bone resorption comprises zones of increased radiolucency in the calvaria. Histologically, these changes are associated with significant periosteal bone resorption on the dorsal calvarial surface and increased marrow spaces, accompanied with increased numbers of osteoclasts. FR167653 significantly reduced the radiographic bone resorption areas (p<0.05; Figure 6) and osteoclast number (as determined on histological sections) (p<0.005, Figure 7).

### Treatment with FR167653 has the effect on the cotnrol of PTHrP-induced systemic hypercalcemia

**Figure 8:**
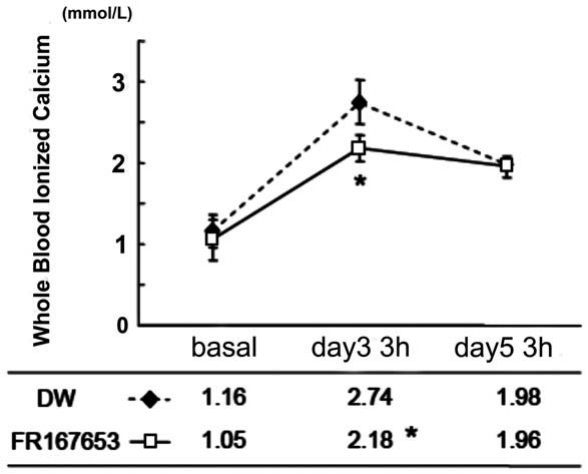
The effect of FR167653 on whole-blood ionized calcium levels in PTHrP-treated mice. PTHrP induces a significant elevation of whole-blood ionized calcium levels in mice within three hours of injection with PTHrP (at day three and five). The whole-blood ionized calcium levels are lower in the FR167653-treated group than in the DW-treated group (control group) at day three (*: P<0.05). By day five, however, there was no significant difference between two groups.

PTHrP is one of the causative factors of the hypercalcemia that is associated with malignancy, since PTHrP can induce systemic bone resorption. Furthermore, extensive local bone resorption is to some extent a contributory cause of hypercalcemia. For this reason, we monitored whole-blood ionized calcium levels in our experimental system. The level of whole-blood ionized calcium was increased at three hours after PTHrP injection on days 3 and 5 compared to day 1. When additionally treated with FR167653, the whole-blood ionized calcium concentration was significantly decreased at day 3, but not day 5. This suggest the functional involvement of p38 MAPK in PTHrP-induced systemic hypercalcemia (Figure 8).

## Discussion

There are several reports about p38 MAPK inhibitor, SB203580, inhibits osteoclast differentiation in RAW264 induced by RANKL [28] or in bone marrow cells induced by 1α, 25- (OH)_2_ D_3_ and prostaglandin E_2_ (PGE_2_) [14]. Previously, we reported that M-CSF-dependent sRANKL- and TNFα-induced osteoclast formation in primary bone marrow cells, and collagen-induced arthritis in rats were inhibited by FR167653 [23]. Here we further show that FR167653, a selective inhibitor of p38 MAPK, strongly inhibits osteoclast differentiation in PTHrP-treated bone marrow cell cultures.

We also investigated the mechanism of FR167653 involved in the inhibition of osteoclast formation. Most osteoclastgenesis inducers are thought to stimulate osteoclast formation via up-regulation of RANKL and down-regulation of OPG gene expression as mediated by osteoblast or bone marrow stromal cells [25], [29]. In our study, however, even the highest concentration of FR167653 that was tested (10 µM) has no effect on RANKL and OPG expression by PTHrP-stimulated primary bone marrow stromal cells. These data suggest that p38 MAPK is not involved in the regulation of PTHrP-mediated bone resorption-related functions of osteoblasts, including regulation of RANKL and OPG expression. This is consistent with previous results that SB203580 has no effect on RANKL and OPG expression by primary osteoblast stimulated with 1α, 25- (OH)_2_D_3_ and PGE_2_ [14]. Together, the data suggest that p38 MAPK is involved in the differentiation of osteoclast precursors, rather than in the function of supporting cell such as osteoblasts or bone marrow stromal cells.

However, the current study found that the up-regulation of c-Fos and NFATc1expression treated with RANKL in osteoclast precursor cells (MDBMMs) were inhibited by FR167653 in a dose-dependent manner in both mRNA and protein levels. This strongly suggested the inhibition of osteoclast differentiation by FR167653 is involved in regulating c-Fos and NFATc1 expression in osteoclast precursor cells. The similar results were reported by using SB203580 [30]. These findings, together with the present study, suggest that p38 MAPK-mediated signal propagation down to c-Fos and NFATc1 are of fundamental importance for the differentiation of osteoclast precursors into osteoclasts.

In our animal experiments, local injection of PTHrP on calvaria caused significant bone resorption and systemic hypercalcemia, and increased osteoclast number. Treatment with FR167653 successfully reduced bone resorption and osteoclast number in calvaria, and eventually protected the calvaria from serious osteoclastic dystruction induced by locally administered PTHrP. However, some relieving effect on hypercalcemia was observed. In our hands, inhibition of p38 MAPK by FR167653 significantly decreased the whole-blood calcium level on day 3. However, there is no difference in blood calcium level between the FR167653 treatment group and the control group at day 5.

It was reported that recombinant human OPG is effective in inhibiting bone resorption and hypercalcemia induced by PTHrP *in vivo* [27]. Treatment with chimeric form of OPG in combination with PTHrP (20 µg/day) maintained whole blood ionized calcium levels within the normal range (∼1.20 mmol/l). Even though FR167653 was given subcutaneously, it should have act systemically. The concentration of calcium in extracellular fluids is under control by a complex homeostatic system strictly that includes the parathyroid glands, kidneys, bones, and intestines [31], [32]. An increase of extracellular calcium concentration is sensed by a calcium-sensing receptor (CaR) in the plasma membrane of parathyroid gland cells and the kidney which in turn affects parathyroid hormone, calcitonin, and 1, 25 (OH) _2_D_3_ secretion. Although intracellular signaling systems regulated by the CaR are still in obscurity, recent evidence suggests that MAPK pathways are activated by CaR [33], [34]. For example, calcium homeostasis by angiotensin II in adrenal glomerulosa cells is mediated by activation of p38 MAPK pathways [35]. These observations may help us to understand the surprisingly small effect of p38 MAPK pathway blockade on regulating hypercalcemia induced by PTHrP because the inhibition of p38 MAPK pathway may block CaR signaling pathway as well, which in turn causes reduced reaction to extracellular Ca^2+^ in tissues that express CaR. The detailed mechanism for why inhibition of p38 MAPK partially prevents hypercalcemia even though it is effective in reducing bone resorption merits further studies.

FR167653 has been used in several inflammatory disease models and no obvious adverse events were observed [15]–[19]. In our previous *in vivo* study [23], we found no significant side effects with daily treatments of 32 mg/kg, a dose found to be safe and effective in other inflammation models as well [36]. In the present study, we also found that there is no significant side effects when twice daily treatment with 30 mg/kg FR167653 for about 1 week. Long-term injection of FR167653 may lead to toxic events; indeed, one study demonstrated that FR167653 treatment increased plasma creatine and lactate dehydrogenase levels in rats [37]. Clearly, the potentially adverse effects of FR167653, including its modulation of calcium homeostasis, need to be studied extensively.

In conclusion, our data indicate that a potent p38 MAPK inhibitor, FR167653, blocks PTHrP-induced osteoclastogenesis *in vitro*, and bone resorption and hypercalcemia *in vivo*. Our results indicate that the responses of other tissues or organs to the p38 MAPK inhibitor may affect calcium homeostasis. This study provides a plausible explanation and target for PTHrP-induced osteoclastogenesis, which will help us to understand the mechanism of bone resorption-related diseases.
